# The Protein Acetylation after Traumatic Spinal Cord Injury: Mechanisms and Therapeutic Opportunities

**DOI:** 10.7150/ijms.92222

**Published:** 2024-02-12

**Authors:** Hong-wei Li, Hai-hong Zhang

**Affiliations:** Department of Spine Surgery, Lanzhou University Second Hospital; Orthopaedics Key Laboratory of Gansu Province, Lanzhou 730030, China.

**Keywords:** spinal cord injury, protein acetylation, HAT, HDAC, inhibitors

## Abstract

Spinal cord injury (SCI) leads to deficits of various normal functions and is difficult to return to a normal state. Histone and non-histone protein acetylation after SCI is well documented and regulates spinal cord plasticity, axonal growth, and sensory axon regeneration. However, our understanding of protein acetylation after SCI is still limited. In this review, we summarize current research on the role of acetylation of histone and non-histone proteins in regulating neuron growth and axonal regeneration in SCI. Furthermore, we discuss inhibitors and activators targeting acetylation-related enzymes, such as α-tubulin acetyltransferase 1 (αTAT1), histone deacetylase 6 (HDAC6), and sirtuin 2 (SIRT2), to provide promising opportunities for recovery from SCI. In conclusion, a comprehensive understanding of protein acetylation and deacetylation in SCI may contribute to the development of SCI treatment.

## 1. Introduction

Spinal cord injury (SCI) is a devastating and irreversible disease of the central nervous system (CNS), which can lead to permanent deficits in motor, sensory, and autonomic functioning 1. The most common causes of traumatic SCI are car accidents, sports accidents, broken bones, and gunshot wounds 2-4. The severity of an injury is determined by physical forces, such as compression, shearing, laceration, and acute stretch/distraction 2. Generally, spinal fractures caused by the above factors are often accompanied by SCI, ultimately leading to severe paraplegia, varying degrees of respiratory dysfunction of cervical fractures, and varying degrees of motor and sensory dysfunction below the injury plane due to thoracolumbar fractures.

SCI can be classified into two phases: primary injury and secondary injury 5, 6. Primary SCI occurs in the initial stage immediately after the injury, which includes the disruption of axons, blood vessels, and cell membranes 7. After the primary injury phase, secondary injury has a crucial effect on the pathophysiological process of SCI and is a destructive and processive cascade occurring later with neuronal necrosis and apoptosis, axonal rupture, demyelination, immune inflammatory reaction, and activation of aberrant molecular signaling 8. During the secondary injury cascade, the release of chemicals, such as reactive oxygen, glutamate, and aspartate, leads to neuronal excitotoxicity and additional loss of neurons and glia by both necrotic and apoptotic cell death 9, 10. Furthermore, these chemicals can negatively affect nucleic acid, proteins, and phospholipids around the lesion, resulting in neurological dysfunction 11.

Due to disruption of the blood-spinal cord barrier, various inflammatory cells infiltrate into the injury sites and contribute to the release of inflammatory cytokines such as tumor necrosis factor-α (TNF-α), interleukin (IL)-1α, IL-1β, IL-6, and IL-17 after 6-10 hours following injury 12, 13. Researchers have shown that these cytokines promote necrosis, apoptosis, and neurodegeneration, impeding SCI repair 13. Meanwhile, ion imbalance after SCI aggravates the secondary injury zone, activates calcium-dependent proteases, and triggers mitochondrial dysfunction, which is closely related to the activation of necrotic and apoptotic cell deaths 14. Therefore, more intervention on the pathogenesis of secondary injury can represent a potentially effective therapeutic target to overcome repair failure by SCI.

Recent studies have suggested that epigenetic regulations are involved in the mechanism of gene modification, thus influencing cell growth, differentiation, apoptosis, autophagy, and transformation 15, 16. A variety of diseases arising from inflammation, internal or external damages, alter epigenetic regulations, including DNA methylation, histone methylation, and histone acetylation. Evidence has demonstrated that epigenetic regulations play an essential role in mediating spinal cord plasticity and neuronal differentiation during SCI 17-19. The epigenetic modification processes are associated with mechanisms that underlie the repair and therapy of SCI.

This review focuses on the evidence for modification of protein acetylation in SCI. We summarize the regulating effects of histone and non-histone acetylation and outline possible pharmacological interventions that could be used to modulate and target this acetylation modification in SCI.

## 2. Protein acetylation

Protein acetylation is one of the most common protein modifications. It can be divided into the two major types: co-translational Nα-terminal acetylation and post-translational Nε-lysine acetylation 20, 21. Nα-terminal acetylation occurs on the α-amino group at the N-terminus of proteins and the process is considered to be irreversible 22. Nε-lysine acetylation refers to the acetylation of the ε-amino group of a lysine residue (more typically referred to as lysine acetylation) 21. Nε-lysine acetylation has been thoroughly investigated and is a widespread and versatile regulation form. In this review, acetylation refers only to post-translational Nε-lysine acetylation. It is acetylation that enables proteins to possess abundant activities and functions by modulating protein stability, enzyme activity, subcellular localization, and interactions with other PTMs 23, 24. Because each modified protein is distinct, acetylation can be classified as histone acetylation or non-histone acetylation. Histone acetylation and non-histone acetylation are involved in regulating transcription, autophagy, metabolism, signal transduction, protein folding, differentiation, and neural function 25.

### 2.1 Acetylation-related enzymes

Acetylation is regulated by two types of enzymes: the lysine acetyltransferases (KATs), which catalyze the transfer of an acetyl group from acetyl coenzyme A to the lysine residue on a polypeptide chain, and the lysine deacetylases (KDACs), which catalyze the removal of acetyl group 26, 27. Taking into consideration histone acetylation, KATs and KDATs were customarily called histone acetyltransferases (HATs) and histone deacetylases (HDACs), respectively. Manifold studies firmly demonstrated that apart from histones, acetylation has been implicated in tens of thousands of non-histone proteins in biological processes 28. KATs whose enzymatic activities as well as biological structures and functions were well characterized in mammalians are classified into three main families: the GCN5/PCAF family; the p300/CBP family; and the MYST family 29, 30. These subfamilies of KATs share a conserved central core region that contributes to the acetyl-CoA-binding (KAT) domain 31. The other remaining KATs will not be referred to in this review. Another class of enzymes, KDACs (known as HDACs), are divided into two major subgroups: Zn^2+^-dependent HDACs (HDAC1-11) and NAD^+^-dependent sirtuins (SIRT1-7, among which only SIRT1, SIRT2, and SIRT3 function as strong deacetylases) 32. Besides, KDACs are also divided into four subtypes according to structure and function. Class I HDACs, including HDAC1, HDAC2, HDAC3, and HDAC8, are mainly located in the nucleus. Class II HDACs, which are grouped into class IIa (HDAC4, HDAC5, HDAC7, and HDAC9) and class IIb (HDAC6 and HDAC10), shuttle between the nucleus and cytoplasm. Class III HDACs are NAD^+^-dependent sirtuins.

### 2.2 Histone acetylation

It is well known that acetylation occurs in more than 40 different lysine residues within the four classic core histones, H2A, H2B, H3, and H4, which play a fundamental role in the initiation and elongation of transcriptional regulation 33, 34. This process is dynamically and reversibly regulated by a balance between HATs and HDACs. Research has reported that histone acetylation can regulate gene transcription. For instance, hyper-acetylation of histone H3 on the promoter region of MIP-2 and CXCR2 may lead to augmentation of the MIP-2/CXCR2 axis and induce chronic neuroinflammation 35. Histone acetylation also contributes to the recruitment of chromatin remodeling factors, which are ATP-dependent enzymes capable of sliding, expelling, or modifying nucleosome spacing to expose specific DNA segments 36. Histone acetylation has a profound effect on the response to epileptic insults, cerebral trauma, and neuropathic pain 17, 37, 38.

### 2.3 Non-histone acetylation

With the further study of histone acetylation for more than half a century, researchers have gradually paid attention to non-histone acetylation. Research has shown that non-histone acetylation participates in physiological or pathological processes relevant to various diseases, such as cell growth, autophagy, apoptosis, and DNA damage repair. Acetylation of non-histone is mediated by KATs and removed by KDACs. Non-histone acetylation could affect protein stability, enzymatic activity and subcellular localization of the targeted proteins. For example, studies have demonstrated that the acetylation of transcription factor p53 increases its protein stability, interaction with other proteins, and binding ability to promoters 39, 40. Cell cycle regulators, including cyclin-dependent kinase 1 (CDK1), CDK2, the protein kinases BUBR1, and Aurora kinase A and Aurora kinase B are also modulated by acetylation to influence cell cycle 41, 42. Acetylation of tau by CBP/p300 has been suggested to impair its function and contribute to its pathological aggregation in Alzheimer's disease 43. Another discovery suggested that the kinases AKT and PDK1 in their pleckstrin homology domains are acetylated by p300/CBP-associated factor (PCAF) and p300, obstructing their membrane localization and activation 44. Thus, non-histone acetylation serves as an attractive regulation form and a therapeutic target in the pathophysiological process.

## 3. Histone acetylation after SCI

Histone acetylation and deacetylation act as a mediator of axonal and neuronal regeneration in SCI by modeling developmental gene expression and chromatin remodeling. A study has clarified that acetylation of histone H4 plays an important role in neural plasticity, synaptogenesis, synaptic plasticity, learning, and memory. After SCI, the level of global histone H4 acetylation was time-related and influenced glial fibrillary acidic protein level, thus H4 acetylation can be further used for tissue remodeling after SCI 45. It was confirmed in previous studies that histone H4 acetylation induced by HDAC inhibitors could module a variety of regeneration-associated genes (RAGs) to improve spinal cord plasticity, axonal growth, and sensory addxon regeneration after SCI 46. Research found that there was increased acetylation of histone H3K9 via PCAF at the promoters of growth-associated protein 43 (GAP-43), galanin, and BDNF following a peripheral axonal injury, thus contributing to axonal regeneration 47. Moreover, histone acetylation mediated by Creb-binding protein also increased the expression of genes related to regenerative process and program, further promoting axon regeneration after SCI 48. On the contrary, a paclitaxel-induced neuropathic pain model revealed that enhanced histone H4 acetylation might upregulate the expression of CX3CL1, which is implicated in neuropathic pain following SCI 49. Evidence suggested that the acetylation levels of histone H3 at the promoter of the inflammatory molecules such as MIP-2 and CXCR2 were increasing. The status contributed to the expressions of MIP-2 and CXCR2, thus inducing chronic neuroinflammation and neuropathic pain 35. CBP/p300 exerts a crucial role as a HAT of H3K9 and H4K5 in upregulating the expression of the pro-nociceptive molecules, BDNF and cyclooxygenase-2 in a rat model with chronic constriction injury 50, 51. Taken together, histone acetylation plays a dual role in neuronal development and recovery after SCI. The overview of the role of histone acetylation after SCI is shown in Table 1.

## 4. Non-histone acetylation after SCI

Acetylation of non-histone proteins, including α-tubulin, heat shock protein 90 (HSP90) and p53, plays a fundamental role in axon regeneration and neuron differentiation in SCI. The PTM of these proteins through acetylation refers to various KATs and KDACs, which regulates their interaction with proteins, subcellular distribution, stability, and activity 52. Non-histone acetylation is further discussed to provide new insights into the mechanism of the secondary damage and therapeutic target after SCI. The overview of the role of non-histone acetylation after SCI is shown in Table 1.

### 4.1 Acetylation of α-tubulin after SCI

Microtubules, as the most important component of cytoskeleton, consist of α-tubulin and β-tubulin. They not only affect changes in cell morphology, transports of intracellular substances, and expression of intracellular proteins 53, but also provide environmental information within the organization and energy transfer 54. Microtubules are subject to tubulin acetylation, a crucial epigenetic modification in regulating microtubule structure and stability. The level of tubulin acetylation is mainly mediated by α-tubulin acetyltransferase1 (αTAT1)^55^. On the contrary, the reverse reaction of the process is catalyzed by HDAC6 56, 57. Abnormal level of α-tubulin acetylation is associated with neurological disorders, including SCI. Research has demonstrated that the expression of α-tubulin was decreased after SCI and that remodeling microtubules could repair SCI 58, 59. Targeting α-tubulin acetylation as well as the corresponding enzymes offers a promising insight into SCI.

αTAT1 is a microtubule-specific acetyltransferase that catalyzes α-tubulin Lys40 acetylation and directly affects the stability of microtubules in cells. αTAT1 plays a positive role in promoting axonal growth in vitro by acetylating α-tubulin 60. In addition, elongator protein 3 regulates α-tubulin acetylation to contribute to the migration and differentiation of cortical neurons 61.

HDAC6, a member of the HDAC family, is mainly localized and is actively maintained in the cytoplasm 62. HDAC6 has a dynamic effect on reversible acetylation that the overexpression of HDAC6 contributes to α-tubulin deacetylation, whereas inhibition of HDAC6 increases α-tubulin acetylation. α-Tubulin acetylation was significantly decreased, and this phenomenon was associated with loss of axonal transport in these neurodegenerative diseases, including Alzheimer's disease, Parkinson's disease, and amyotrophic lateral sclerosis 63-65. Targeting inhibition of HDAC6 can recover the normal α-tubulin acetylation levels and axonal transport in these neurodegenerative diseases 66. A study inhibiting HDAC6 via the administration of tubastatin A, a special selectivity compound for HDAC6, certified that the levels of acetylated tubulin were increased and axonal regeneration was promoted 67. In addition, SIRT2 is NAD^+^-dependent deacetylase and is mainly found in the cytoplasm. SIRT2 deacetylates α-tubulin dependent on the CDK5 phosphorylation in neurons 68. A study found that alternatively polarized macrophages 2 (M2) increase SIRT2 expression, which deacetylates α-tubulin in microtubules to further facilitate the differentiation of ependymal stem cells toward neurons following SCI 69.

Therefore, the regulation of α-tubulin acetylation and deacetylation by related enzymes plays a role in neural functional recovery and provides new insights into neurological disease interventions, including SCI.

### 4.2 Acetylation of other non-histone proteins after SCI

HSP90 and its co-chaperones orchestrate essential physiological processes including cell survival, cell cycle, and apoptosis 70. HSP90 acetylation can modulate its interaction with both client and co-chaperone proteins 71. Existing report has demonstrated that HDAC6 is a crucial deacetylase of HSP90 72. HDAC6 regulates chaperone-mediated autophagy to resist hypoxia-ischemia-induced oxidative stress by affecting HSP90 acetylation after SCI 73. HSP90 acetylation may play a role in neuroprotection and cell antioxidant activity after SCI. Another study found that the acetylation of p53 via CBP/p300 promotes axon outgrowth and regeneration by augmenting the GAP-43 expression 74.

## 5. Target therapy

In this review, we further discuss the target treatment involved in HDAC inhibitors and HAT activator in various neurological diseases, including SCI. The overview of the therapies targeting protein acetylation after SCI is shown in Table 2.

### 5.1 HDAC inhibitors

A growing body of literature has suggested that HDAC inhibitors exert indispensable effects on neuroprotection and anti-inflammation in the CNS 75. For example, CI-994, known as a benzamide-based class I HDAC inhibitor, has effects on neuroprotection and contributes to functional recovery by targeting HDAC1 and HDAC3 in an experimental model of SCI 76, 77. Consistent with this, tubastatin A, which has a pharmacological inhibitory effect on HDAC6, promotes acetylation of tubulin and partially repairs autophagic flux impaired by SCI, thus facilitating axon regeneration and functional recovery after SCI 67. According to one study, HDAC inhibitors, such as MS-275 and MGCD0103, reduce behavioral hypersensitivity and alleviate neuropathic pain in a rat model of traumatic nerve injury 78. Another research has demonstrated that trichostatin A (TSA) inhibits the protein expression of HDAC2 through promoting ubiquitination-mediated degradation of HDAC2, and thus plays an analgesic effect in nerve injury 79. Valproic acid (VPA) exerts various therapeutic advantages in diseases, including epilepsy, SCI, and cancers 80-82. Recent evidence has suggested that VPA, as a HDAC inhibitor, mediates high acetylation of the STAT1/NF-κB pathway by suppressing HDAC3 expression and activity. As a result, acetylation of the complex with NF-κB p65 and STAT1 inhibited the transcriptional activity of NF-κB p65 and attenuated the central inflammatory response mediated by microglia after SCI 83. Moreover, sodium valproate, a global HDAC inhibitor, contributes to anti-hypersensitivities in rats by mediating glutamate transporters following peripheral nerve injury 84.

### 5.2 HAT activator

In a chronic SCI model, pharmacological activation of CBP/p300 by CSP-TTK21 enhances histone acetylation and the expression of RAGs. Furthermore, CSP-TTK21 triggers motor and sensory axon growth, sprouting, and synaptic plasticity, suggesting that it may be a molecular regenerative intervention to recover from SCI 85.

## 6. Conclusions

The last two decades have witnessed tremendous advances in clarifying the molecular mechanisms and targeting therapies for SCI. Acetylation of histone and non-histone proteins has been extensively and deeply studied in many diseases, and it plays an important role in various biological processes and development of diseases. Many enzymes, including αTAT1, HDAC6, and SIRT2, are not only involved in protein acetylation and deacetylation, but also maintain a balance between the two. Studies have provided substantial evidence suggesting that epigenetic modification through protein acetylation regulates gene transcription, signal transduction, and interaction with other developmental proteins after SCI. Protein acetylation excerts a crucial role in SCI and becomes the entry point for SCI treatment. Targeting protein acetylation-related enzymes, such as HAT activators and HDAC inhibitors, can provide new ideas for SCI treatment as well as new therapeutic targets for axon growth and regeneration.

However, the current research on the epigenetic modulation of SCI has several limitations. First, targeting protein acetylation primarily focuses on the level of animal models and rarely involves humans. Second, the role of protein acetylation differs according to the time points after SCI, making it difficult to determine the optimal time to intervene. Finally, SCI recovery requires the involvement of multiple regulatory mechanisms, of which protein acetylation is only one. Thus, the development of single-cell sequencing, high-resolution quantitative mass spectrometry, and selective chemical probes aids in defining specific regulatory genes and precise mechanisms.

Although complete recovery is difficult for patients with SCI, therapeutic strategies targeting epigenetic regulation can reduce the pain caused by SCI and enhance neuronal repair as much as possible. With the advancement of cell transplantation and gene therapy, it is promising to target protein acetylation modification to recover from SCI.

## Figures and Tables

**Table 1:** Overview of the major roles of protein acetylation after SCI.

Type of modification	Modified enzymes	Specific substrates	State of acetylation after SCI	Major roles	References
Histone acetylation		histone H4	Increase	Improve spinal cord plasticity, axonal growth, and sensory addxon regeneration after SCI	45, 46
	PCAF	histone H3	Increase	Contribute to axonal regeneration	47
		histone H4	Increase	Implicated in neuropathic pain following SCI	49
		histone H3	Increase	Induce chronic neuroinflammation and neuropathic pain	35
	CBP	histone H3 and H4	Increase	Promote axon regeneration after SCI	50, 51
Non-histone acetylation	αTAT1	α-tubulin Lys40	Increase	Affect the stability of microtubules; Promote axonal growth in vitro	60, 62, 68
	HDAC6	α-tubulin	Decrease	Inhibition of HDAC6 to recover the normal α-tubulin acetylation levels and axonal transport	66
	CBP/p300	P53	Increase	Promote axon outgrowth and regeneration	74
	HDAC6	HSP90	Decrease	Resist hypoxia-ischemia-induced oxidative stress after SCI	72, 73

**Table 2:** The overview of the therapies targeting protein acetylation after SCI.

HDAC inhibitors or HAT activator	Substrate	Proposed functions	References
CI-994	HDAC1 HDAC3	Neuroprotection and contribute to functional recovery	76, 77
tubastatin A	HDAC6	Promote acetylation of tubulin; Facilitate axon regeneration and functional recovery from SCI	67
MS-275 MGCD0103	HDAC1	Reduce behavioral hypersensitivity and alleviate neuropathic pain	78
trichostatin A (TSA)	HDAC2	Play an analgesic effect in nerve injury	79
Valproic acid (VPA)	HDAC3	Attenuate the central inflammatory response mediated by microglia after SCI	80-82
CSP-TTK21	CBP/p300	Promote axon growth, sprouting, and synaptic plasticity	85
